# High expression of WTAP leads to poor prognosis of gastric cancer by influencing tumour‐associated T lymphocyte infiltration

**DOI:** 10.1111/jcmm.15104

**Published:** 2020-03-16

**Authors:** Huafu Li, Qiao Su, Bo Li, Linxiang Lan, Chunming Wang, Wuguo Li, Gangqiang Wang, Wei Chen, Yulong He, Changhua Zhang

**Affiliations:** ^1^ Digestive Medicine Center The Seventh Affiliated Hospital of Sun Yat‐Sen University Shenzhen China; ^2^ Department of Gastrointestinopancreatic Surgery The First Affiliated Hospital of Sun Yat‐Sen University Guangzhou China; ^3^ Adult Stem Cell Laboratory The Francis Crick Institute London UK; ^4^ Animal Experiment Center The First Affiliated Hospital Sun Yat‐Sen University Guangzhou China

**Keywords:** differentially expressed genes, DNA methylation, gastric cancer, N6‐methyladenosine (m6A) methylation, WTAP

## Abstract

**Background:**

N6‐methyladenosine (m6A) methylation, a well‐known modification with new epigenetic functions, has been reported to participate in gastric cancer (GC) tumourigenesis, providing novel insights into the molecular pathogenesis of GC. However, the involvement of Wilms’ tumour 1‐associated protein (WTAP), a key component of m6A methylation, in GC progression is controversial. Here, we investigated the biological role and underlying mechanism of WTAP in GC.

**Methods:**

We determined WTAP expression using tissue microarrays and The Cancer Genome Atlas (TCGA) data set, which was used to construct co‐expression networks by weighted gene co‐expression network analysis (WGCNA). Gene Ontology (GO) and Kyoto Encyclopedia of Genes and Genomes (KEGG) enrichment analyses were performed by Database for Annotation, Visualization and Integrated Discovery (DAVID). CIBERSORT was used to determine WTAP expression in 22 immune cell types.

**Results:**

Wilms’ tumour 1‐associated protein was highly expressed in GC, which indicated a poor prognosis, and WTAP expression served as an independent predictor of GC survival. By WGCNA, GO, KEGG and core gene survival analyses, we found that high WTAP expression correlated with RNA methylation and that low expression correlated with a high T cell–related immune response. CIBERSORT was used to correlate low WTAP expression with T lymphocyte infiltration.

**Conclusion:**

RNA methylation and lymphocyte infiltration are the main causes of high WTAP expression and poor prognosis, respectively.

## 1 INTRODUCTION

Gastric cancer (GC) is one of the most common malignant tumours of the digestive system, and it has a high proportion of malignant tumour‐related mortality worldwide, especially in China. According to statistical analysis, GC was ranked 5th in the global incidence of malignant tumours in 2018, but the mortality rate was ranked 3rd.^1^ Research suggests that the causes of GC are related to genetic factors, stomach diseases, *Helicobacter pylori* and lifestyle.^2^ Although the treatment of GC has made great progress in recent decades, ranging from interventional therapy or radical resection to targeted therapy or immunotherapy, the treatment results of GC are still not ideal.^3^ Therefore, it is necessary to further clarify the molecular mechanism of GC to develop new therapeutic strategies to reduce the mortality of this malignant tumour.

N6‐methyladenosine (m6A) methylation modifications are one of the most common methylation modifications in eukaryotes. It accounts for more than 80% of RNA methylation, and its modification site always appears in the conserved sequence RRACH (R = G or A, H = A, C or U).^4^ The mammalian Wilms’ tumour 1‐associating protein (WTAP) is the first nuclear protein associated with the Wilms’ tumour 1 inhibitor gene WT1, discovered by Little et al.^5^ Wilms’ tumour 1‐associating protein (WTAP) is a component of the m6A methyltransferase complex that recruits the m6A methyltransferases METTI3 and METTL14 to the corresponding mRNA targets to co‐catalyse the formation of m6A.^6^ Deregulation of m6A pathway components can affect oncogenic expression, thereby affecting tumourigenesis.^7^ Since most studies have focused on the intrinsic carcinogenic pathways of tumours, the potential role of mRNA m6A modification in host antitumour immune responses remains unclear. Dali Han et al studied the mechanism of the antitumour effect of the mRNA m6A methylation gene YTHDF1 and found that dendritic cells regulate the methylation of mRNA m6A through YTHDF1 and thus play a role in antitumour immunity.^8^ WTAP is a component of the m6A methyltransferase complex, and the potential role of WTAP in host antitumour immune responses is unclear, so we need to explore it further. To this end, we studied the expression of WTAP in GC tissue, the effect of WTAP expression on tumour immune cell infiltration and patient prognosis, and then explored the mechanism by weighted gene co‐expression network analysis (WGCNA).

## 2 PATIENTS AND METHODS

### 2.1 Study subjects

A total of 14 GC patients were recruited from the First Affiliated Hospital of Sun Yat‐sen University, including 9 males and 5 females. The average age was 54.93 ± 7.42 years old, and the age range was 44‐68 years old. The study was approved by the Ethics Committee of the First Affiliated Hospital of Sun Yat‐sen University. The samples were all obtained with the informed consent of the patients. Surgical pathological staging criteria were in accordance with the International Disease for Oncology (ICD‐O): four cases in stage I and stage II, 10 cases in stage III and stage IV; degree of differentiation: three cases of moderate differentiation, two cases of poor differentiation, seven cases of medium‐low differentiation and two cases of high differentiation (see Table 1).

**Table 1:** Demographics of stomach carcinoma patients

	SYSU	TCGA	*P*
Male	9	204	.833
Female	5	114	
Age	(54.93 ± 7.42)	(65.87 ± 10.55)	.089
Pathological staging			
Stage I And II	4	146	.823
Stage III And IV	10	172	
Differentiation			
High	2	7	.966
Moderately	3	113	
Poorly	7	191	
Un‐differentiation	2	7	

The pathology downloaded from the TCGA website (https://portal.gdc.cancer.gov/) in September 2019 was clearly diagnosed as transcriptome and clinical data of patients with gastric adenocarcinoma, including data from 416 cases of gastric adenocarcinoma and general information corresponding to the case. Data without a survival time listed were removed and included 416 cases of GC and 33 cases of adjacent tissues. Inclusion criteria were as follows: (a) diagnostic age ≥ 8 years; (b) tumour site: stomach; and (c) cases with clear pathology. The exclusion criteria were as follows: (a) multisource tumours; (b) carcinoma in situ; (c) incomplete follow‐up information; (d) cases of death within 30 days; and (e) the WTAP expression level is unknown. Finally, 318 patients with GC were included in the survival analysis, including 204 males and 114 females. The average age was 65.87 ± 10.55 years old, and the age range was 35‐90 years old. There were 146 cases in stage I and stage II and 172 cases in stage III and stage IV. The degree of differentiation was 113 cases of moderate differentiation, seven cases of poor differentiation, 191 cases of moderate‐to‐low differentiation and seven cases of high differentiation (see Table 1).

### 2.2 RNA extraction and RT‐qPCR

The mRNA was extracted by TRIzol homogenate from each cancer tissue and its corresponding adjacent tissues, and the concentration was determined. Five micrograms of each sample RNA, 1 µL oligo d(T), 1 µL dNTP and an appropriate amount of DEPC·H2O were added to a total volume of 12 µL and mixed well. The whole system was bathed in a 65°C water bath for 10 minutes, and 4 µL 5x first‐strand buffer, 2 µL 1 µmol/L DTT, 1 µL RNase In and 1 µL M‐MLV were added. The reverse transcriptase was then inactivated by placing in a 75°C water bath for 5 minutes. The cDNA was amplified using primers. The PCR conditions were as follows: pre‐denaturation at 95°C for 5 minutes, then denaturation at 60°C for 20 seconds, annealing at 60°C for 20 seconds and extension at 72°C for 30 seconds for 40 cycles and, finally, 95°C for 15 seconds. The above reagents and primers were supplied by TaKaRa Corporation (TaKaRa Biotech). mRNA expression was detected by ABIS Step One real‐time fluorescence quantitation (ABI Biotech). The amount of relative mRNA expression was calculated by the 2^−ΔΔct^ method for GAPDH normalization (primer sequence see Table 2).

**Table 2:** WTAP primer sequence

Primer name	Primer sequence	Product length/bp
WTAP	F:5′‐GCCAACTGCTGGCGTGTCT‐3′	213
	R:5′‐ATGGCGAAGTGTCGAATGCT‐3′	
GAPDH	F:5′‐GACCCCTTCATTGACCTCAA‐3′	226
	R:5′‐TGCTTCACCACCTTCTTGAT‐3′	

### 2.3 WGCNA co‐expression network construction

Gene expression data (mRNA‐seq data) were obtained from the TCGA database. A total of 24 991 genes were identified from each sample. Analysis of variance was performed, and the data were sorted from large to small. We calculated the standard deviation values for each gene, sorted them from large to small and then selected the top 5000 genes for WGCNA. The expression data map of these 5000 genes was constructed into a gene co‐expression network using the WGCNA package in R software.^9^ Using the WGCNA function adjacency, an adjacency matrix is constructed by computing the Pearson correlation between all pairs of genes in the selected sample. In this study, β = 7 (no scale *R* ^2^ = .9) was used as a soft threshold parameter to ensure a scale‐free network. To further identify the functional blocks in the co‐expression network of the 5000 genes, a topological overlap measure (TOM) is computed using the adjacency matrix, which represents the overlap in the shared neighbourhood.

### 2.4 Identification of clinically significant modules

We identified the relevant modules by calculating the correlation between MEs and WTAP expression levels. The log10 transformation of the p‐value (GS = lgP) in the linear regression of gene expression and clinical WTAP expression is then defined as gene significance (GS). In addition, module significance (MS) is defined as the average GS of all genes in a module. In general, among all selected modules, the module with the highest absolute value of MS is considered to be a module related to the level of WTAP expression.

### 2.5 PPI network construction of key module genes

The hub gene, which is highly interconnected with the nodes in the module, is considered to have important functions. We selected the top 30 hub genes in the module network as candidate genes for further analysis and validation. The STRING data set (https://string-db.org/) is an online biological resource that decodes the interaction between proteins to obtain the true functionality of real proteins.^10^ The candidate gene was submitted to STRING for protein interaction, and the confidence interval for the cut‐off value was set to 0.4. In the Plugin Molecular Complex Detection (MCODE), significance models with strong protein‐protein linkages were calculated and selected with default parameters (degree cut ≥ 2, node score cut ≥ 2, K‐core ≥ 2, maximum depth = 100). The difference was statistically significant at *P* < .05.

### 2.6 Gene Ontology (GO) and pathway enrichment analysis

The Database for Annotation, Visualization and Integrated Discovery (DAVID) is a database for gene function annotation and visualization.^11^ GO and the Kyoto Encyclopedia of Genes and Genomes (KEGG) analyses were performed using the DAVID (version 6.8; https://david.ncifcrf.gov/) online tool (functional annotation tool) for pathway analysis of core genes. The GO results contained three categories: biological processes (BP), cellular components (CC) and molecular functions (MF). Enriched GO terms and KEGG pathways were determined according to the adjusted critical criterion *P* < .05.

### 2.7 Survival analysis of hub genes

Kaplan‐Meier's plotted network (http://kmplot.com/analysis/) is a platform containing expression data for 10 tumour genes and clinical survival data for 1065 patients with GC. We used this website to obtain information on core gene expression and patient survival prognosis, which in turn helped us identify core genes that influence the survival of WTAP high‐ and low‐expression groups.^12^ To assess the prognostic value of a particular gene, patient samples were divided into two groups based on the median expression of the gene (high expression vs low expression). We used a Kaplan‐Meier survival map to analyse the overall survival (OS) of GC patients and uploaded the core genes to the database to obtain the Kaplan‐Meier survival map. The 95% confidence intervals, p‐values and hazard ratios (HRs) were calculated and displayed in the graph.

### 2.8 Enumeration of haematopoietic cell subsets from gene expression profiles

Using normal gene expression data, the number of immune osmotic cells was inferred by calculating the relative proportions of 22 different genotypes using the CIBERSORT algorithm. For the TCGA data set, voom (observation level variance model) was used to convert the RNA sequencing data and the count data to values closer to the microarray results.^13^ The 22 cell types inferred by CIBERSORT included B cells, T cells, natural killer cells, macrophages, dendritic cells, eosinophils and neutrophils. CIBERSORT is a deconvolution algorithm that uses a data set that is considered to represent the smallest expression for each cell type and corresponds to the reference gene expression value (the 547 genes of the "characteristic matrix") based on these algorithms, inferring the proportions of most cell types in tumour samples.^14^ CIBERSORT uses Monte Carlo sampling to obtain a p‐value for the deconvolution of each sample, providing an interval metric for the result. Gene expression data sets were written using standard annotation files and data uploaded to the CIBERSORT web portal (http://cibersort.stanford.edu/), which uses a default feature matrix to run in 1000 permutations. We used a heat map to show the tumour immune cell infiltration in different individual patients. By analysing the correlation between immune cells in different mutation groups, we mapped the correlation heat map. To explore the differences in tumour immune infiltrating cells in different mutant groups, we mapped the violin map visually.

### 2.9 Statistical analyses

The difference in WTAP expression between GC tissues and adjacent tissues was determined by Pearson's chi‐squared test. OS was calculated by survival analysis. OS is defined as the time from the initial diagnosis of primary GC to death for any reason. The survival rate was statistically analysed by Kaplan‐Meier analysis, and the difference in survival curves was statistically analysed by log‐rank test. Spearman's rank correlation (rs) was used to investigate the relationship between tumour immune infiltrating cells among different mutation groups. All statistical analyses were performed using IBM SPSS Statistical Software version 20.0 (IBM Corp.) and R version 3.3.0 (The R Foundation). The P‐value was bilateral, and *P* < .05 was considered statistically significant.

## 3 RESULTS

### 3.1 WTAP was dysregulated in GC

**Figure 1:**
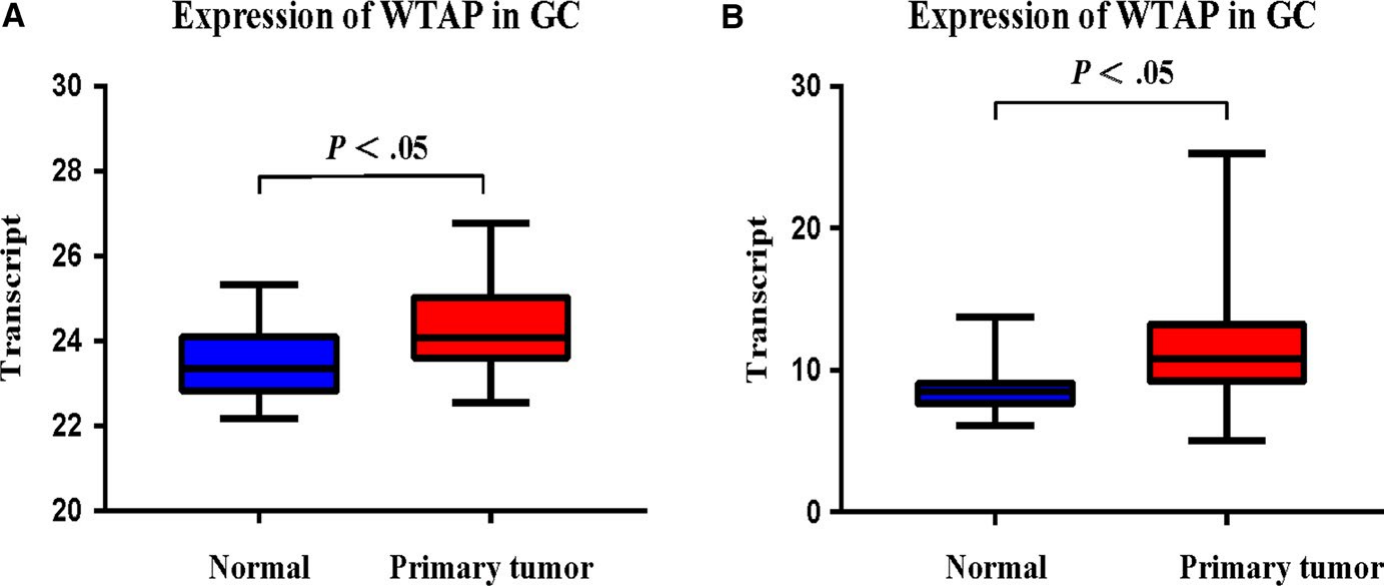
A, WTAP mRNA expression in the pairs of GC tissues and matched adjacent tissues of SYSU Cohort patient. B, WTAP mRNA expression in the pairs of GC tissues and matched adjacent tissues of TCGA Cohort patient

**Figure 2:**
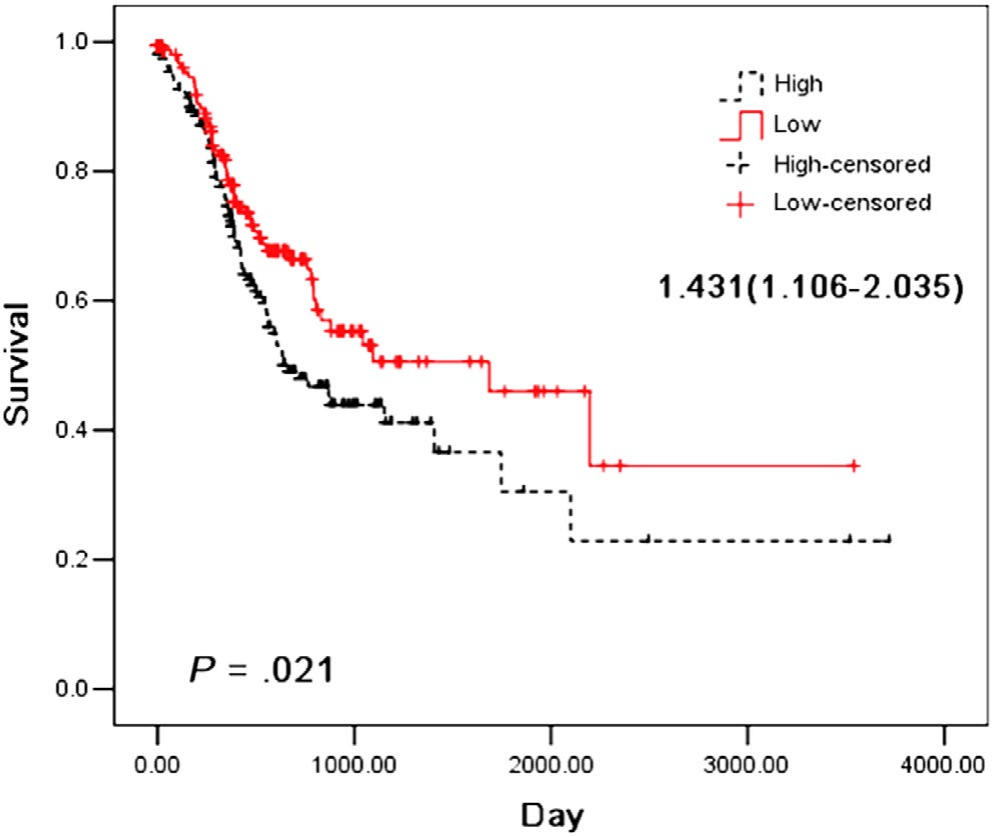
Kaplan‐Meier survival curves of overall survival in TCGA GC patients based on WTAP mRNA expression. The log‐rank test was used to compare differences between two groups

To elucidate the role of WTAP, we first analysed the mRNA expression of WTAP in human GC samples from TCGA data. The results showed that WTAP expression in tumour tissues was significantly increased (Figure 1A). WTAP expression levels were also significantly up‐regulated in GC tissues of our central patients (Figure 1 B). In addition, we also studied the relationship between WTAP expression and survival prognosis in 318 patients with GC. WTAP expression was significantly associated with survival outcomes in patients (Figure 2).

### 3.2 Construction of weighted co‐expression network and identification of key modules

**Figure 3:**
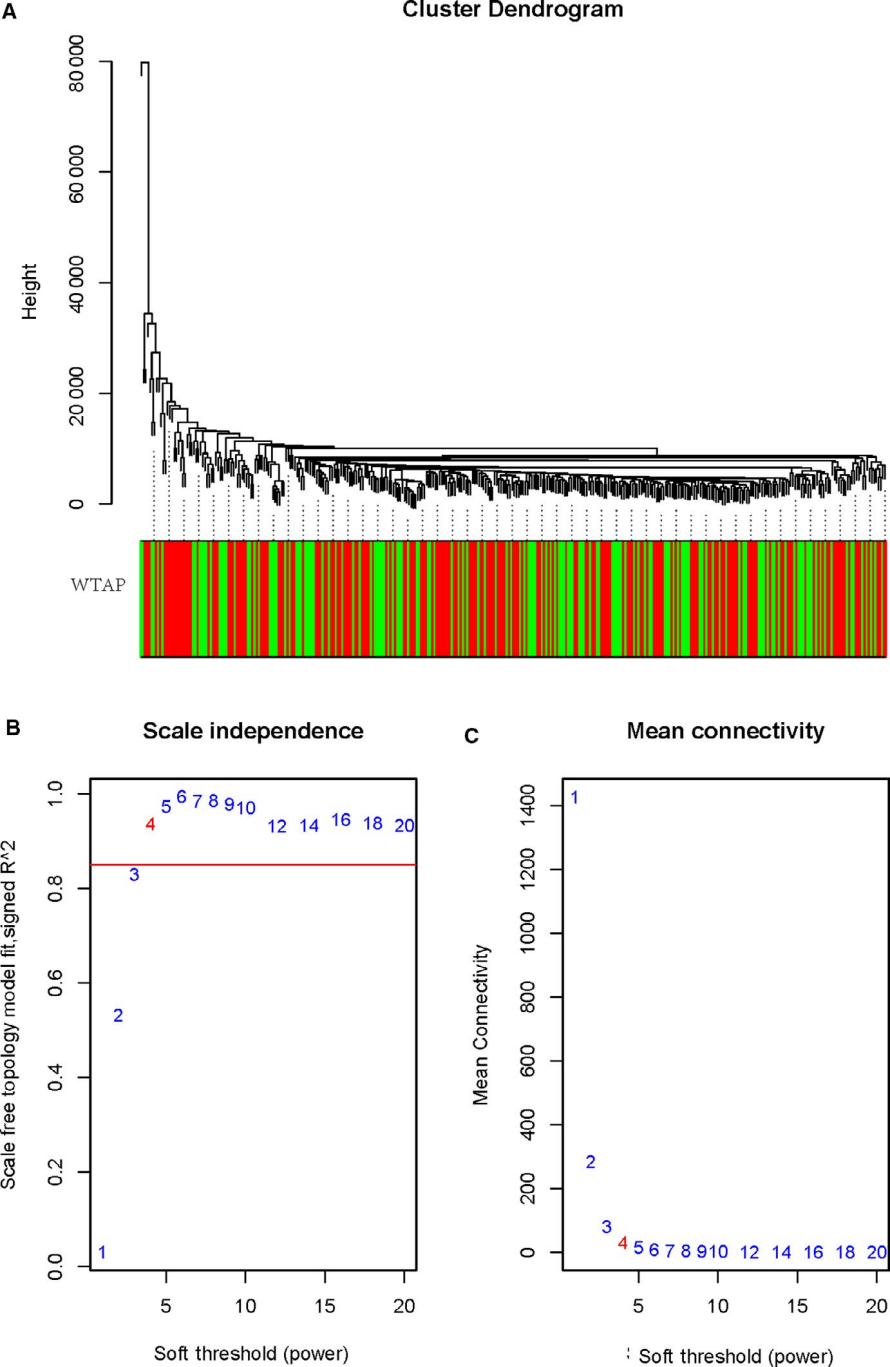
Clustering of samples and determination of soft‐thresholding power. A, The clustering was based on the expression data of TCGA. The top 5000 genes with the highest sD values were used for the analysis by Wgcna. The colour intensity was proportional to expression status (WTAP low and WTAP high). B, Analysis of the scale‐free fit index for various soft‐thresholding powers (β). C, Analysis of the mean connectivity for various soft‐thresholding powers. In all, 4 was the most fit power value

**Figure 4:**
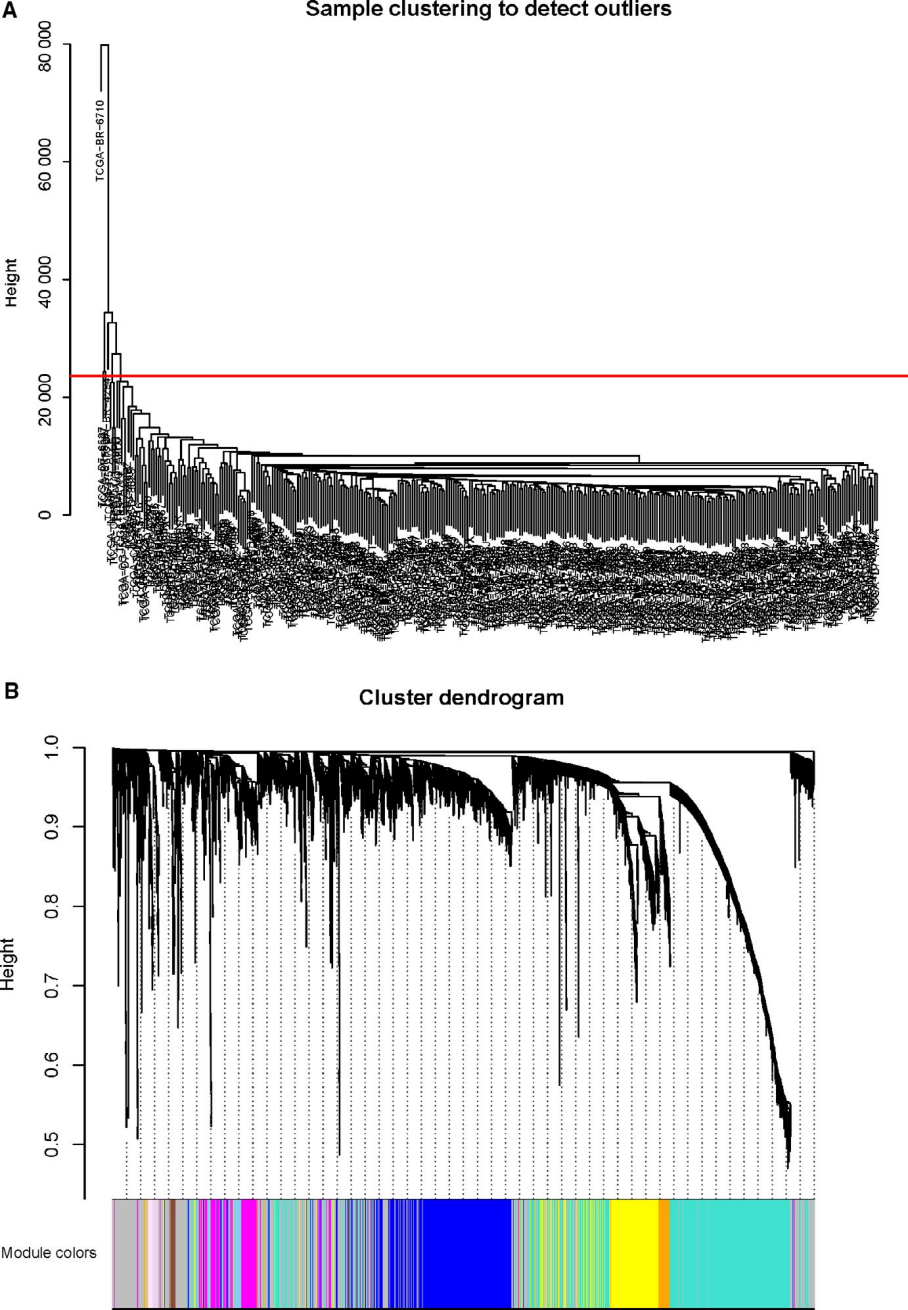
Construction of co‐expression modules by Wgcna package in r. A, The cluster dendrogram of module eigengenes. B, The cluster dendrogram of genes in TCGA. Each branch in the figure represents one gene, and every colour below represents one co‐expression module

To construct a gene co‐expression network, the raw data of GC were downloaded from the TCGA database. The background correction and normalization were performed using R, and the same preprocessing was performed on the original data. R‐pack annotation matching was performed on the probe and the gene symbol, and the probe matching the plurality of genes was removed. For the plurality of probe‐matched genes, the median was taken as the final expression value. Finally, we obtained a total of 24 991 genes. We calculated the standard deviation value for each gene, sorted the values from large to small and then selected the top 5000 genes for WGCNA. Cluster analysis of 5000 genes was performed using the fashClust function of the WGCNA package (Figure 3A). The selection of soft threshold power is an important step in constructing WGCNA. The network topology of 1 ~ 20 threshold weights was analysed, and the scale independence and average connectivity of WGCNA relative equilibrium were determined. As shown in Figure 3B,C, a power value of 9 was selected as the lowest power (0.9) of the scale‐free topology ft index, and a hierarchical clustering tree (dendrogram) of 5000 genes was generated. We set MEDissThres to 0.25 to merge similar modules (Figure 4A) and generated 65 modules (Figure 4B). The gene statistics in each module are shown in Table 3. Genes that cannot be included in any module were added to the grey module and rejected in subsequent analyses.

**Table 3:** Gene statistics in each module

Module	Genes	Module	Genes	Module	Genes	Module	Genes
Antiquewhite4	35	Dark slate blue	51	Maroon	44	Salmon4	45
Bisque4	52	Dark turquoise	119	Medium orchid	32	Sienna3	75
Black	359	Floral white	60	Mediumpurple3	64	Sky blue	98
Blue	997	Green	385	Midnight blue	193	Skyblue2	32
Brown	591	Green yellow	233	Navajowhite2	45	Skyblue3	72
Brown4	54	Grey	2379	Orange	109	Steel blue	86
Coral1	36	Grey60	164	Orangered4	68	Tan	208
Coral2	33	Honeydew1	37	Pale turquoise	85	Thistle1	46
Cyan	194	Ivory	61	Palevioletred3	45	Thistle2	48
Dark Green	122	Lavenderblush3	38	Pink	359	Turquoise	1409
Dark Grey	117	Light cyan	177	Plum1	70	Violet	84
Dark Magenta	75	Lightcyan1	62	Plum2	49	White	99
Dark Olive green	78	Light green	153	Purple	250	Yellow	572
Dark Orange	107	Lightpink4	43	Red	363	Yellow green	74
Darkorange2	54	Lightsteelblue1	64	Royal blue	147		
Darkred	126	Light yellow	151	Saddle brown	95		
Darkseagreen4	36	Magenta	342	Salmon	203		

### 3.3 Correlation between modules and identification of key modules

**Figure 5:**
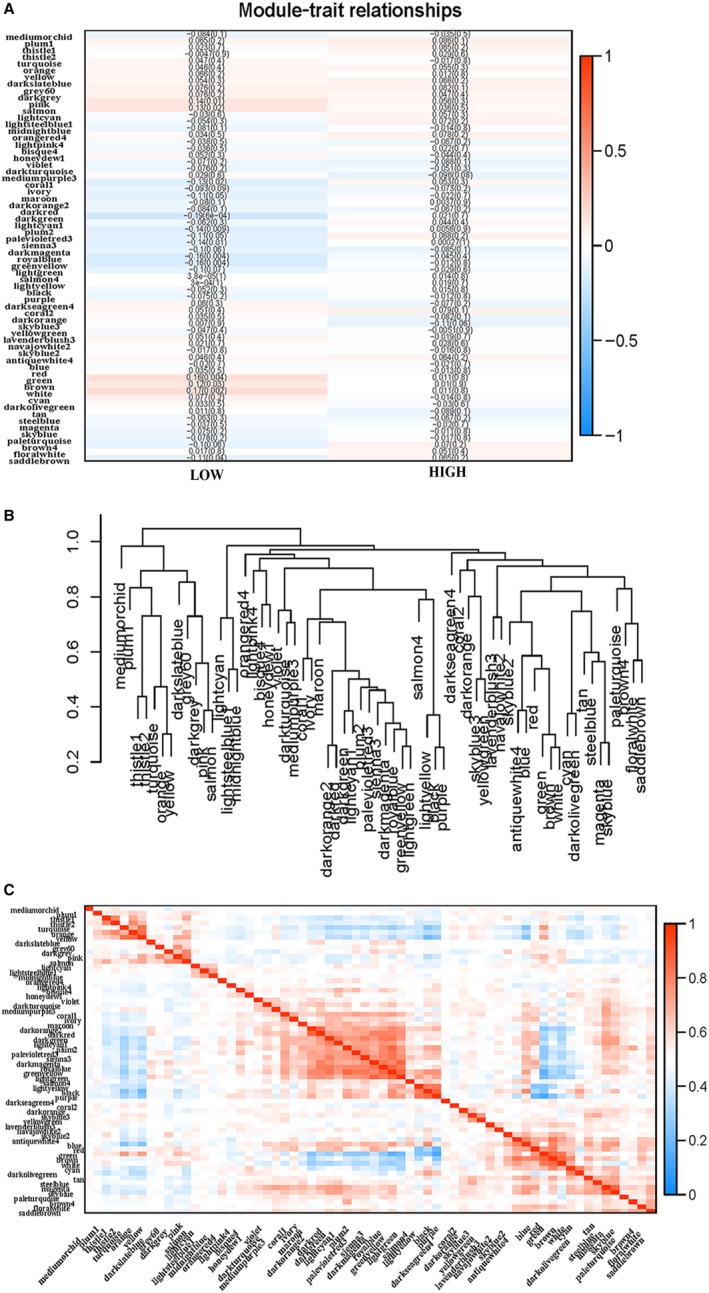
A, Heat map of the correlation between module eigengenes and the expression status of WTAP. The dark orange module was the most positively correlated with WTAP high expression, and the salmon module was the most negatively correlated with WTAP high expression. B, Hierarchical clustering of module hub genes that summarize the modules yielded in the clustering analysis. C, Heat map plot of the adjacencies in the hub gene network

**Figure 6:**
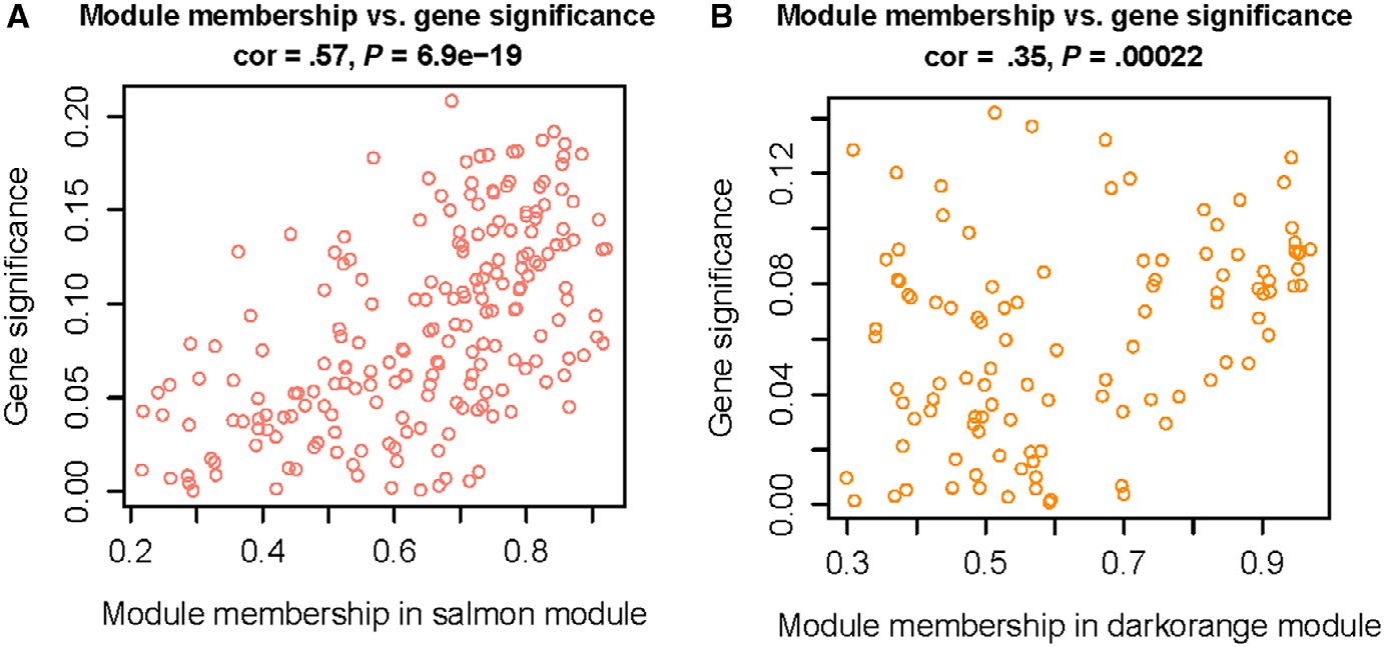
A, Scatter plot of module eigengenes in the salmon module. B, Scatter plot of module eigengenes in the dark orange module

We analysed the interaction between the 65 modules and plotted the network heat map (Figure 5A). The results show that each module is independent of each other, each module has high independence, and the gene expression of each module is relatively independent. In addition, we calculated the characteristic genes and clustered them according to their correlation to explore the co‐expression similarity of all modules (Figure 5B). We found that these 65 modules are mainly divided into two clusters. A heat map drawn from the adjacency relationship shows similar results (Figure 5C). The Salmon module was positively correlated with low WTAP expression, while the dark orange module was positively correlated with high WTAP expression. Figure 6A,B shows the relationship between the number of module members and the GS in the Salmon module and the dark orange module, respectively.

### 3.4 Identification of hub genes in the Salmon module and dark orange module

**Figure 7:**
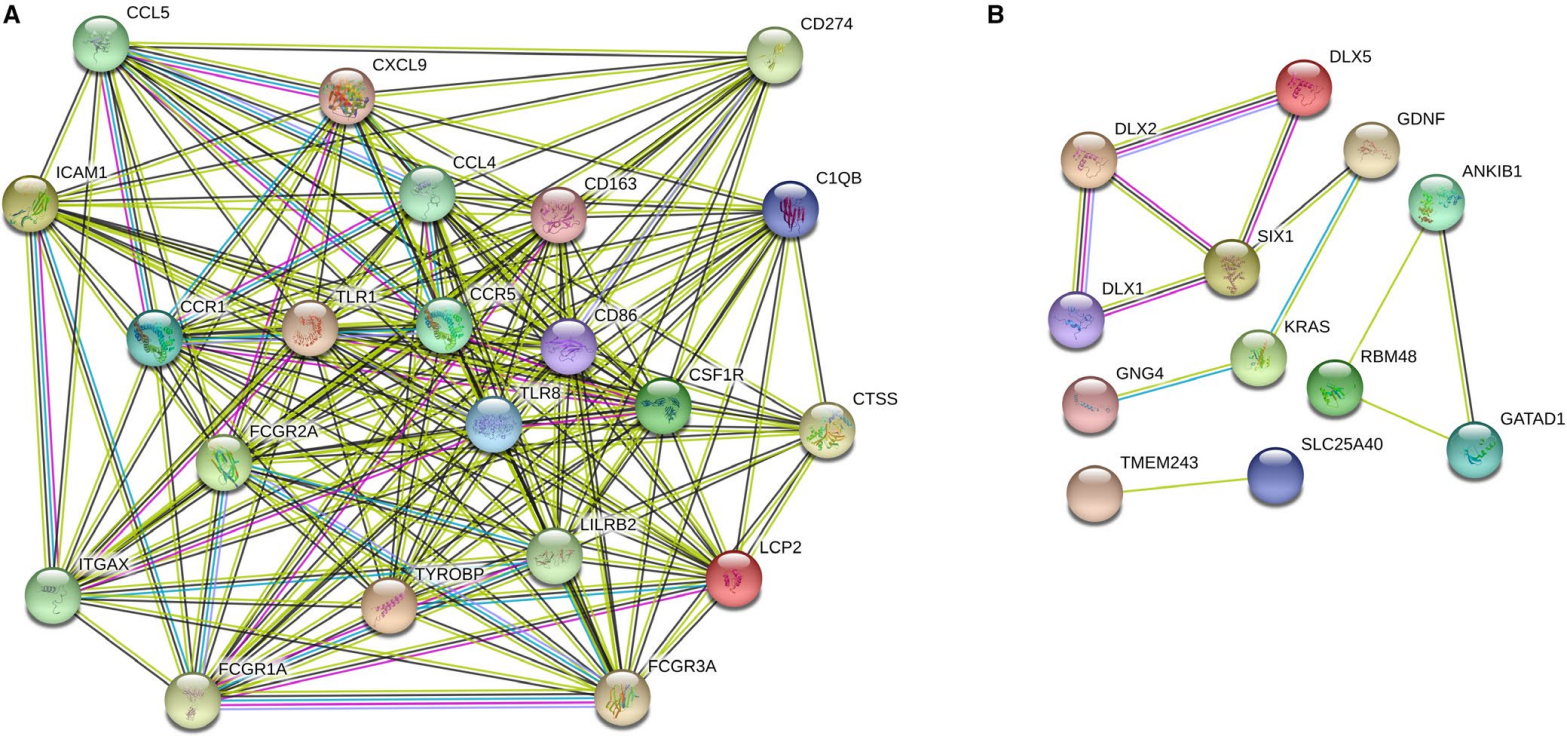
A, The top hub genes in the salmon module. B, The top hub genes in the dark orange module. Edges represent protein‐protein associations. Cambridge blue: from curated databases. Violet experimentally determined. Green gene neighbourhood. Red gene fusions. Blue gene co‐occurrence. Reseda text mining. Black co‐expression. Lilac protein homology

We submitted the gene set of the Salmon module and dark orange module to STRING protein interaction analysis, and the cut‐off confidence interval was set to 0.4. In the Plugin MCODE, significant models with strong protein‐protein linkages were calculated and selected with default parameters (degree cut ≥2, node score cut ≥2, K‐core ≥2, maximum depth = 100). The difference was statistically significant at *P* < .05. The core genes were screened for further analysis by sorting the node degree candidate genes. Figure 7A,B shows the hub genes in the Salmon module and dark orange modules. The core genes of the Salmon module are C1QB, CCL4, ICAM1, CXCL9, CD274, CTSS, CCL5, TLR1, CCR1, CD163, FCGR1A, FCGR2A, FCGR3A, LCP2, CSF1R, LILRB2, ITGAX, TLR8, CCR5, CD86 and TYROBP. The core genes of the dark orange module are ANKIB1, DLX1, GATAD1, GNG4, HOXC9, TMEM243, DLX2, DLX5, KRAS, RBM48, SIX1, SLC25A40, GDNF, HOXB5, HOXC6 and HOXC8.

### 3.5 Functional enrichment analysis in the two key modules

To study the roles of the core genes in these two key modules, we conducted an enrichment analysis and explored the pathways involved in BPs. The GO enrichment of BPs by DAVID showed that the Salmon module was mainly enriched in immune responses such as immune response, defence response, cellular defence response, inflammatory response, response to wounding and cell surface receptor linked to signal transduction (Figure S1, S2 and S3A). The dark orange module was mainly enriched in embryonic skeletal system morphogenesis, epithelial cell differentiation, cell fate commitment, anterior/posterior pattern formation, epithelial development, positive regulation of transcription from RNA polymerase and other types of expression regulation (Figure S1, S2 and S3B).

### 3.6 Survival analysis of the core gene

Genes with significant interactions in the WTAP low‐expression group included C1QB, CCL4, ICAM1, CXCL9, CD274, CTSS, CCL5, TLR1, CCR1, CD163, FCGR1A, FCGR2A, FCGR3A, LCP2, CSF1R, LILRB2, ITGAX, TLR8, CCR5, CD86 and TYROBP. Genes with significant interactions in the WTAP high‐expression group included ANKIB1, DLX1, GATAD1, GNG4, HOXC9, TMEM243, DLX2, DLX5, KRAS, RBM48, SIX1, SLC25A40, GDNF, HOXB5, HOXC6 and HOXC8. To further investigate the impact of core genes on patient outcomes, we used a combination of expression levels to study the impact of the core genes on prognosis. The genes associated with prognosis in the WTAP low‐expression group were CCR1, CD163, FCGR1A, FCGR2A, FCGR3A, LCP2, CSF1R, LILRB2, TLR8, CCR5 and CD86 (Figure S4). The expression of these genes is significantly associated with poor prognosis in patients. The genes associated with prognosis in the WTAP high‐expression group are DLX2, DLX5, KRAS, RBM48, SIX1, SLC25A40, GDNF, HOXB5, HOXC6 and HOXC8 (Figure S5). The expression of these genes is clearly related to the prognosis of patients.

### 3.7 Immune infiltration in different WTAP expression groups

**Figure 8:**
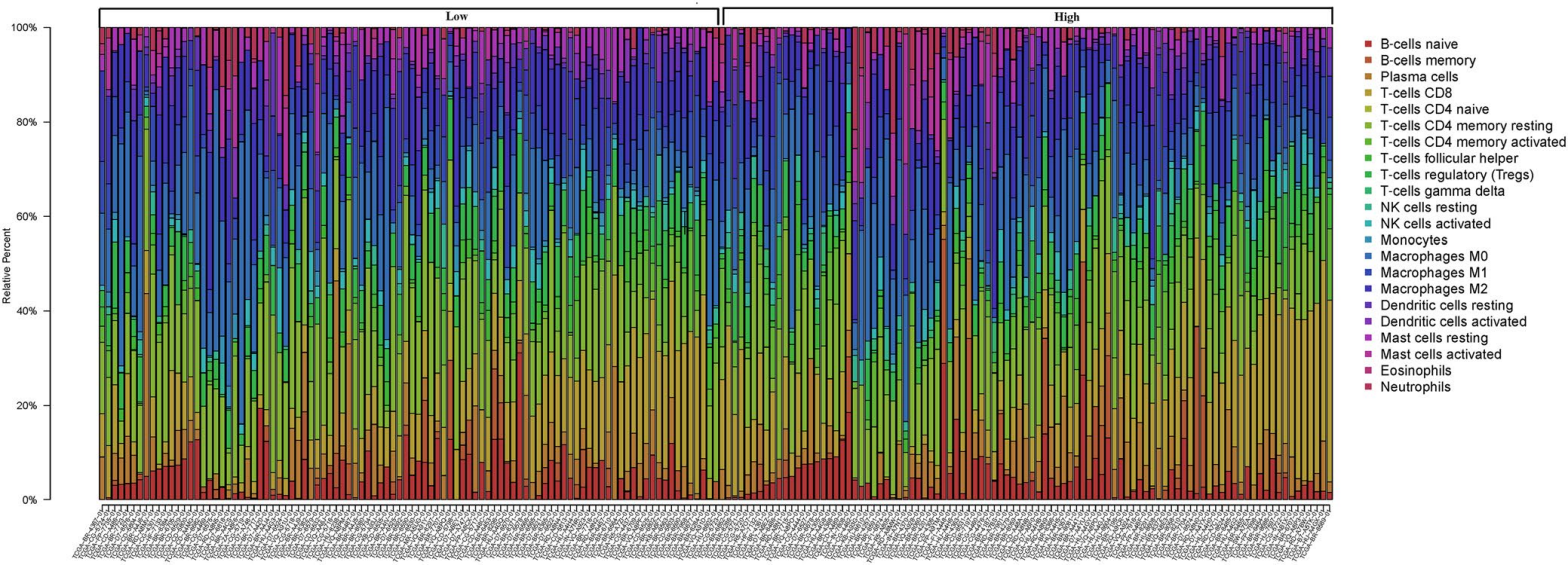
The proportion of immune cell subsets in the WTAP high‐expression group and WTAP low‐expression group

**Figure 9:**
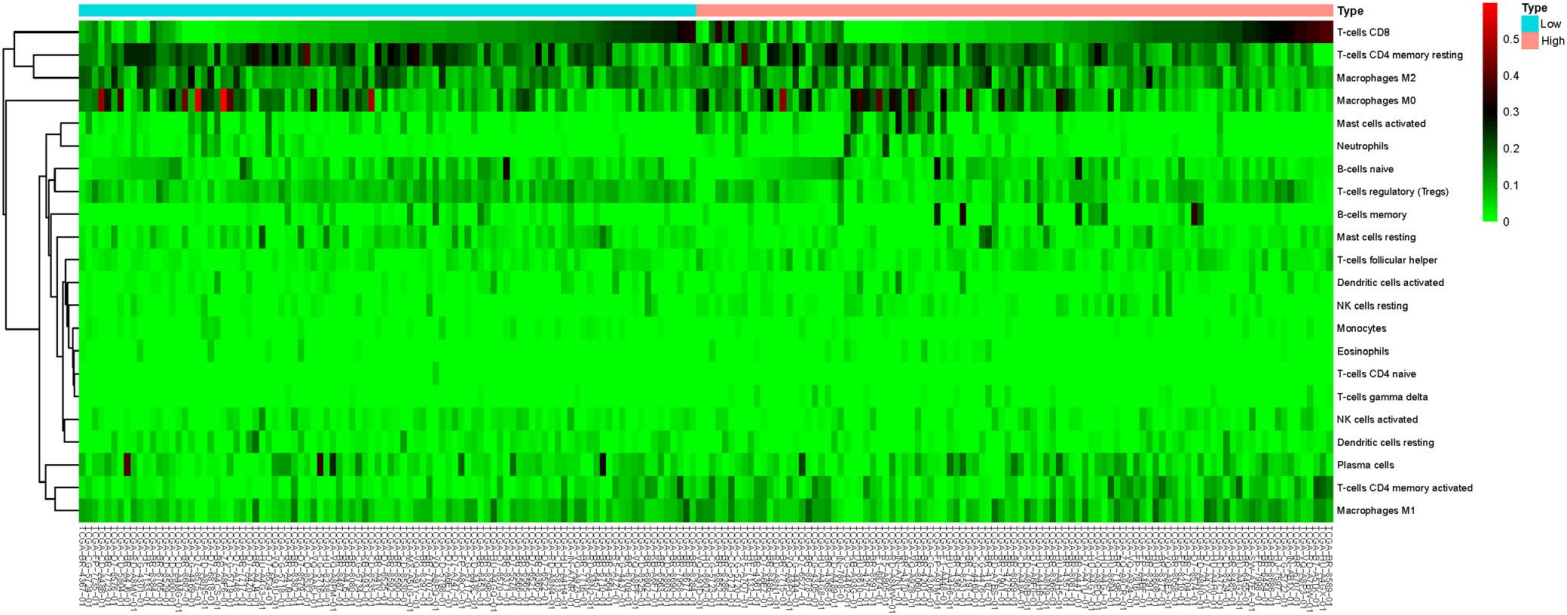
Heat map of different immune cell subsets in WTAP high‐expression group and WTAP low‐expression group

**Figure 10:**
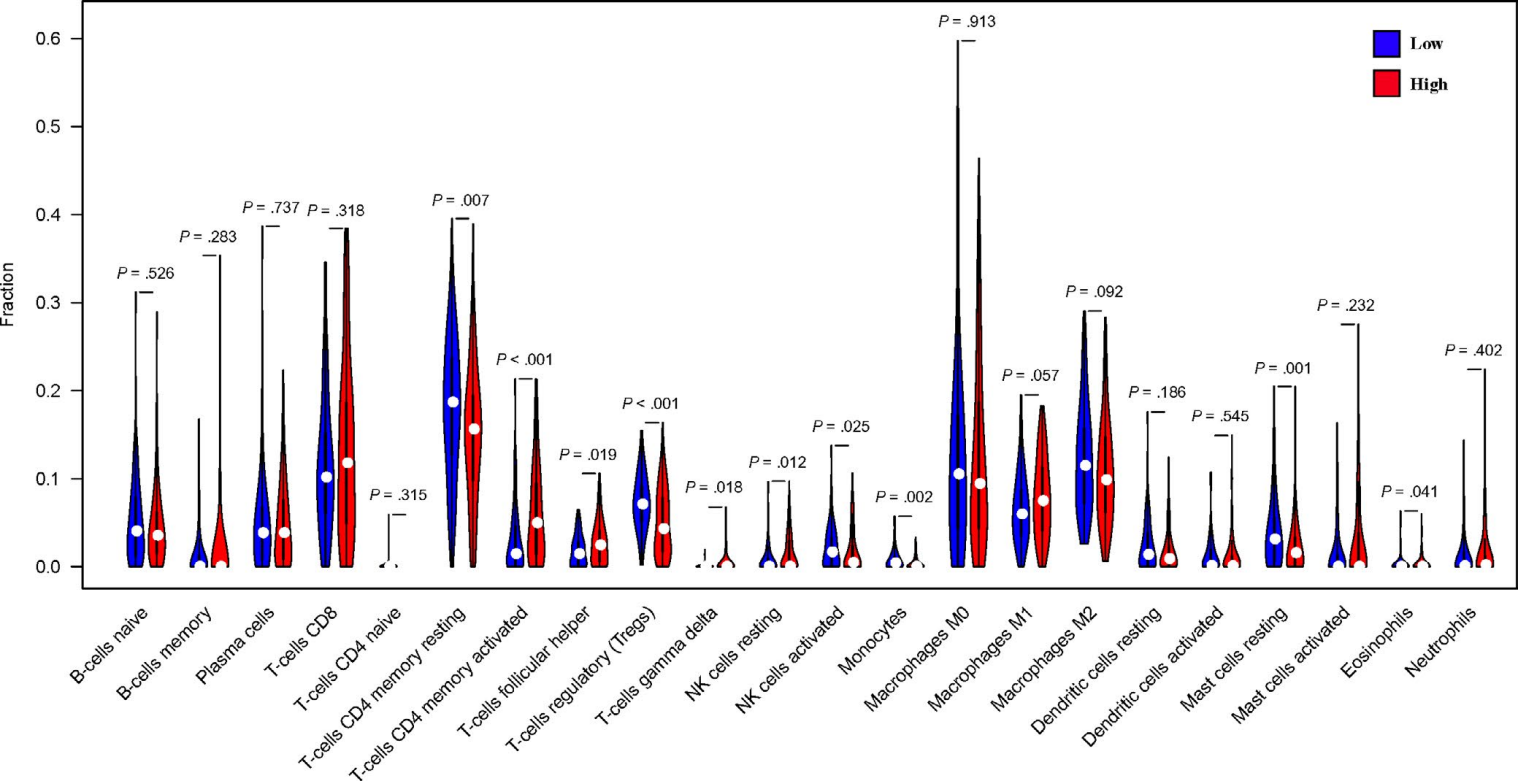
The violin map of the statistical differences between the tumour cells of different WTAP expression groups

**Figure 11:**
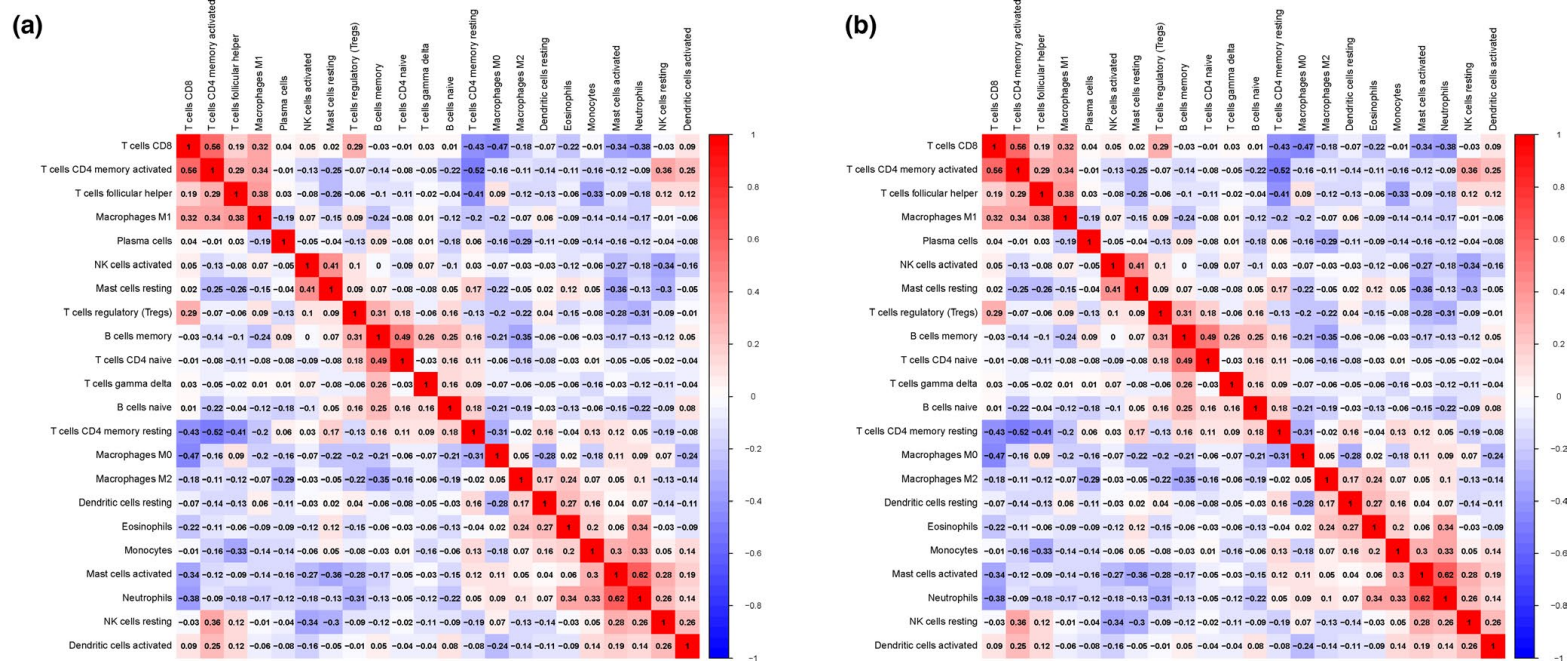
A, The correlation heat map of the correlation between WTAP high expression group tumor immunoinfiltrating cells. B, The correlation between WTAP low expression group tumor immune infiltrating cells

By comparing the proportion of immune cell subsets in the different WTAP expression groups, we can see that the proportion of resting CD4 memory cells, regulatory T cells (Tregs), activated NK cells, monocytes and resting mast cells in the WTAP low‐expression group was significantly higher than that in the high‐expression group (*P* < .05). The proportion of CD4 memory‐activated T cells and follicular helper T cells in the WTAP high‐expression group was significantly higher than that in the low‐expression group (*P* < .05) (see Figures 8, 9 and 10). To explore the correlation between invasive immune cells in different mutant groups of GC, we compared the correlation between differential immune infiltrating cells by different WTAP expression groups. In Figure 11, it can be seen that there is a significant difference in the correlation between tumour immune infiltrating cells between the different WTAP expression groups. In the WTAP low‐expression group, resting CD4 memory was negatively correlated with CD4 memory activation, regulatory T cells (Tregs) were negatively correlated with B‐cell memory, and activated NK cells were positively correlated with resting mast cells (Figure S6A). For the WTAP high‐expression group, CD4 memory‐activated T cells were positively correlated with CD8 T cells and follicular helper T cells (Figure S6B). To explore the effects of these cell infiltrations and prognosis, we conducted a comparative analysis and found that high infiltration of regulatory T cells (Tregs) and CD4 memory‐activated T cells can improve patient outcomes (Figure S7).

## 4 DISCUSSION

m6A RNA modification is a hotspot in the field of regulation in recent years, involving multiple cellular processes such as mRNA maturation, protein translation and molecular structure transformation.^15^ There is growing evidence that m6A dysregulation has a profound impact on the pathogenesis of many diseases, including GC.^16^ We examined the expression of WTAP in GC samples and found that WTAP expression in tumour tissues was higher than that in adjacent tissues. The data from the TCGA database also confirmed our conclusions. To investigate whether the expression of WTAP has an effect on the prognosis of patients, we found that patients with high expression of WTAP have a poor prognosis and poor patient expression. This shows that the impact of WTAP in GC is distinctive.

WGCNA is a method for constructing gene co‐expression networks based on gene expression data.^17^ To explore the molecular mechanisms that influence the effect of WTAP expression on prognosis, we used WGCNA to find the core co‐expressed genes. The core genes expressed under low WTAP expression were C1QB, CCL4, ICAM1, CXCL9, CD274, CTSS, CCL5, TLR1, CCR1, CD163, FCGR1A, FCGR2A, FCGR3A, LCP2, CSF1R, LILRB2, ITGAX, TLR8, CCR5, CD86 and TYROBP. The genes associated with the prognosis of the core genes of the WTAP low‐expression group were CCR1, CD163, FCGR1A, FCGR2A, FCGR3A, LCP2, CSF1R, LILRB2, TLR8, CCR5 and CD86 (Table 4). The expression of these genes is significantly associated with poor prognosis in patients. Through enrichment analysis, these genes were found to enhance the role of tumour immune cells. The core genes highly expressed by WTAP are ANKIB1, DLX1, GATAD1, GNG4, HOXC9, TMEM243, DLX2, DLX5, KRAS, RBM48, SIX1, SLC25A40, GDNF, HOXB5, HOXC6 and HOXC8, among which the core genes and prognosis of the WTAP high‐expression group are DLX2, DLX5, KRAS, RBM48, SIX1, HOXB5, HOXC6 and HOXC8 (Table 4). The expression of these genes is clearly related to the prognosis of patients. Through enrichment analysis, these genes were found to up‐regulate the methylation of mRNA. Studies have found that m6A RNA modifications can affect tumour proliferation and patient prognosis through immunoregulatory effects.^18^ Our study also found that T regulatory cells (Treg) and CD4 memory‐activated T cells in patients with high WTAP expression were significantly lower than those in patients with low WTAP expression. There is a clear correlation between the infiltration of these cells and the prognosis of patients. This suggests that tumour immune regulation may be an important cause of poor prognosis in WTAP.

**Table 4:** Known function/phenotype of protein‐protein interaction analysis of differential genes in different WTAP expression of gastric cancer

Protein	Protein function	Expression
CCR1^28^	Encodes a member of the beta chemokine receptor family, whose ligands include macrophage inflammatory protein 1 (MIP‐1) alpha, regulated on activation normal T expressed. The high expression of CCR1 is significantly related to better prognosis of tumour.	WTAP low
CCR5^28^	Encodes a member of the beta chemokine receptor family, which is similar to G protein‐coupled receptors. CCR5 is expressed by T cells and macrophages. The high expression of CCR5 is significantly related to better prognosis of tumour.	WTAP low
CD163^29^	CD163 in a soluble form in plasma, it has an anti‐inflammatory function. The high expression of CD163 is significantly related to better prognosis of tumour.	WTAP low
CD86^29^	A membrane protein present on some germinal‐centre B cells, mitogen‐activated B cells and monocytes that serves as a B‐cell activator. The high expression of CD86 is significantly related to better prognosis of tumour.	WTAP low
FCGR1A, FCGR2A, FCGR3A^29^	FCGR1A, FCGR2A and FCGR3A are also known as CD64, CD32 and CD16. CD64, CD32, CD16, CD163 and CD86 are homologous leucocyte differentiation antigens. The high expression of FCGR1A, FCGR2A and FCGR3A is significantly related to better prognosis of tumour.	WTAP low
LCP2^31^	A gene which acts as an adaptor or scaffold protein may play a role in T‐cell development and activation and in mast cell and platelet function. The relationship between this gene and tumour, and the tumour‐related effects is not yet clear.	WTAP low
CSF1R^32^	The CSF1R gene provides instructions for making a protein called the colony stimulating factor 1 receptor (CSF‐1 receptor). The high expression of CSF1R is significantly related to better prognosis of tumour.	WTAP low
LILRB2^33^	This gene is found in a gene cluster at chromosomal region 19q13.4. The relationship between this gene and tumour, and the tumour‐related effects is not yet clear.	WTAP low
TLR8^34^	The gene which plays a fundamental role in pathogen recognition and activation of innate immunity. The high expression of LILRB2 is significantly related to better prognosis of tumour.	WTAP low
DLX2^35^, DLX5^36^	DLX2 has been shown to interact with DLX5. The expression of DLX2 is significantly related to the development and prognosis of tumour.	WTAP high
SIX1^37^	The genes in a family provide instructions for making proteins that bind to DNA and control the activity of other genes. The expression of SIX1 is significantly related to the development and prognosis of tumour.	WTAP high
HOXB5^38^, HOXC6^39^, HOXC8^40^	This gene is regulation of mRNA expression and affects transcription and expression through expression regulation. The relationship between this gene and tumour, and the tumour‐related effects is not yet clear.	WTAP high
RBM48^41^	RNA‐binding protein 48. Regulation of mRNA expression by binding to related proteins. The relationship between this gene and tumour, and the tumour‐related effects is not yet clear.	WTAP high
KRAS^42^	This gene, a Kirsten ras oncogene homolog from the mammalian ras gene family, encodes a protein that is a member of the small GTPase superfamily. The expression of KRAS is significantly related to the development and prognosis of tumour.	WTAP high

In fact, WTAP overexpression is an important risk factor in many tumours.^19^ WTAP is widely expressed in various tissues and plays an important role in cell cycle regulation, RNA alternative splicing, m6A methylation modification, x‐chromosome inactivation, eye development, regulation of physiological balance and other physiological processes.^20^ The role of WTAP in GC is currently rarely reported and controversial. Zhang C et al found that low m6A levels can lead to the proliferation of GC cells, resulting in GC progression.^21^ Wang et al studied the related effects of m6A methylation on the prognosis of GC and found that m6A methylation can lead to the progression of GC and can lead to poor prognosis of GC patients.^22^ We studied the effect of WTAP on the prognosis of GC and found that high WTAP expression was associated with poor prognosis of patients. To explore this cause, our study found for the first time that WTAP can affect the prognosis of GC patients by regulating the roles of immune cells. Winkler et al found that m6A can act as a negative regulator of the type I IFN response by controlling IFNA and IFNB mRNA expression.^23^ IFNs stimulate the JAK/STAT pathway and activate the expression of hundreds of inactivated genes (ISGs) against viral infections.^23^ The conflicting effects of type I IFN signalling on immune responses are well recognized. Interferon, type I interferon and IFN‐γ (IFNG) promote SAR and CD8 + T‐cell activity and T‐cell cross‐priming.^24^ Multiple studies have shown that type I interferons (IFNs) play an important role in tumour control by promoting the reactivation (reactivation) of T cells by dendritic cells (DCs).^25^ Our study found that high expression of WTAP can reduce T‐cell infiltration and inhibit tumour immunity, which may be supported by the above studies. Liang et al found that IFNs may be an important target for PDL1 immunotherapy, and they also showed that methylation genes are a potential target for this research.^26^

The study of methylation genes in immune regulation is currently a hot issue, and WTAP plays an indelible role as an important member of the methylation genes.^27^ However, WTAP is poorly understood in the study of immune regulation. This study found for the first time that WTAP expression is significantly associated with T lymphocyte infiltration and that WTAP expression can inhibit the production of immune cells. The roles of CCR1, CD163, FCGR1A, FCGR2A, FCGR3A, LCP2, CSF1R, LILRB2, TLR8, CCR5 and CD86 and the core genes of immunoregulation were also confirmed by WGCNA. There is increasing evidence that chemokines play a role in promoting the growth, survival and metastasis of GC.^28^ CCR1 and CCR5 are active in the family of chemokine receptors, and CCR5 and its ligands may be involved in the activation of T cells. This system is related not only to the immune response, inflammation and viral infection but also to the occurrence and spread of tumours.^28^ FCGR1A, FCGR2A and FCGR3A are also known as CD64, CD32 and CD16. CD64, CD32, CD16, CD163 and CD86 are homologous leucocyte differentiation antigens that are able to activate T‐cell proliferation through antigen‐antibody reactions.^29^ Studies have also shown that CD86 and CD163 have a significant correlation with the proliferation and progression of GC.^30^ LCP2,^31^ CSF1R,^32^ LILRB2^33^ and TLR8^34^ both interact with T lymphocytes in tumour immune regulation and affect tumour prognosis. These core genes in patients with low WTAP expression enhance the function of tumour immunity, conferring patients with low WTAP expression with a better survival prognosis and better gene expression levels. Conversely, these results also indicate that high WTAP expression may have an effect on tumour immunosuppression. DLX2,^35^ DLX5,^36^ SIX1,^37^ HOXB5,^38^ HOXC6,^39^ HOXC8,^40^ RBM48 41 and KRAS 42 are genes involved in the regulation of mRNA expression and transcription. m6A is an important type of RNA modification. WTAP is a component of the m6A methyltransferase complex. It recruits the m6A transferases METTI3 and METTL14 to the corresponding mRNA targets for co‐catalysis and m6A formation.^43^ It is present in ribosomal RNA (rRNA), transport RNA (tRNA), messenger RNA (mRNA) and non‐coding RNA (ncRNA). m6A is the most widely distributed methylation modification in eukaryotic mRNA, and its formation may modulate a series of processes after transcription, such as splicing, transport, degradation and translation of pre‐mRNA. The formation of m6A is catalysed by a large methyltransferase complex.^44^ Our study also found that high WTAP expression in patients can affect the splicing, transport, degradation and translation of mRNAs by interacting with the DLX2, DLX5, SIX1, HOXB5, HOXC6, HOXC8, RBM48 and KRAS core genes.

## 5 CONCLUSION

Through this study, it was found that the expression of WTAP was significantly correlated with the survival prognosis of patients. To determine the mechanism, we used WCGNA and enrichment analysis to confirm that high WTAP expression is associated with RNA expression, while low WTAP expression is associated with lymphocyte infiltration. This is also the main cause of high WTAP expression and poor prognosis. Further, we found that lymphocyte infiltration in patients with low WTAP expression has a good correlation with patient prognosis. However, this study has certain limitations and deficiencies. First, due to the lack of certain data in the TCGA database, this study did not provide a good analysis of clinical parameters and prognosis. Second, we only analysed the transcriptome levels from patients and did not perform further in vivo and in vitro experiments. Further research is needed to support our conclusions.

## CONFLICT OF INTEREST

All authors declare that they have no conflicts of interest.

## AUTHOR CONTRIBUTIONS

CZ and HL conceived and designed the study. CW, WC, LW, GL and LL performed the data analysis. HL, QS and BL wrote the paper. All authors read and approved the manuscript.

## ETHICS APPROVAL AND CONSENT TO PARTICIPATE

Our research was approved by the ethics committee of the First Affiliated Hospital of Sun Yat‐Sen University, and we obtained written informed consent from all these patients.

## Data Availability

The authors confirm that the data supporting the findings of this study are available within the article and its supplementary.
